# Supplementary material for: Neutral, Negative, or Negligible? Changes in Patient Perceptions of Disease Risk Following Receipt of a Negative Genomic Screening Result
Source: J Pers Med. 2020 Apr 17;10(2):24. doi: 10.3390/jpm10020024 (PMC7354612; doi:10.3390/jpm10020024)
Supplement: Supplementary file 1 [file jpm-10-00024-s001.pdf]

Table S1. Text of letter used to return negative results to participants

Thank you for participating in the RAVE (Return of Actionable Variants Empirical) study.

As you may recall, in this study you gave us permission to sequence your genetic material (DNA) for multiple disease-relevant genes using stored blood sample from the [Mayo Clinic Biobank/Mayo Clinic Vascular Disease Biorepository]. The genes sequenced included those related to:

- high cholesterol levels
- risk of colon cancer
- other genetic conditions for which intervention is possible

Your results: **No clinically significant genetic variants were found. No further action on your part is needed at this time.**

Your complete test report is included in this mailing. A copy of this report will be scanned into your Electronic Health Record. Additionally, your Primary Care Provider at Mayo Clinic will be notified of your results.

Please note that there were some limitations to our testing:

1. We did not test all genes that can cause disease. Even though no genetic variants were identified, you may still be at-risk for the listed conditions.

2. Even in genes that were tested, our genetic testing cannot detect all variants that may eventually be known to cause disease. Future research may find more disease-causing variants.

3. If you have a family history of the listed conditions, or if you have concerns for your own health with regard to these conditions, you may choose to discuss them with your Primary Care Provider.

We thank you for your participation in this research study. Please know that even though we did not find any clinically significant genetic variants in your sample, your results are still valuable to this research study.

If you have questions about this study or your results, please call xxx-xxx-xxxx. If you wish, we can set up a time for you to talk to a genetic counselor to further discuss your results.
